# Self-harm in children 12 years and younger: characteristics and outcomes based on the Multicentre Study of Self-harm in England

**DOI:** 10.1007/s00127-021-02133-6

**Published:** 2021-07-19

**Authors:** Galit Geulayov, Debbie Casey, Liz Bale, Fiona Brand, Ellen Townsend, Jennifer Ness, Muzamal Rehman, Keith Waters, Caroline Clements, Bushra Farooq, Nav Kapur, Keith Hawton

**Affiliations:** 1 Centre for Suicide Research, Department of Psychiatry, Warneford Hospital, University of Oxford, Oxford, UK; 2 Oxford Health NHS Foundation Trust, Oxford, UK; 3 Self-Harm Research Group, School of Psychology, University of Nottingham, Nottingham, UK; 4 Centre for Self-Harm and Suicide Prevention Research, Derbyshire Healthcare NHS Foundation Trust, Derby, UK; 5 Centre for Suicide Prevention, Manchester Academic Health Sciences Centre, University of Manchester, Manchester, UK; 6 Greater Manchester Mental Health NHS Foundation Trust, Manchester, UK

**Keywords:** Self-harm, Children, Adolescents, Methods, Socioeconomic disadvantage

## Abstract

**Background:**

Very little is known about self-harm in children. We describe the characteristics and outcomes of children under 13 years who presented following self-harm to five hospitals in England.

**Methods:**

We included children under 13 years who presented after self-harm to hospitals in the Multicentre Study of Self-harm in England. Information on patients’ characteristics and method of self-harm was available through monitoring of self-harm in the hospitals. Area level of socioeconomic deprivation was based on the English Index of Multiple Deprivation (IMD).

**Results:**

387 children aged 5–12 years presented to the study hospitals in 2000–2016, 39% of whom were 5–11 years. Boys outnumbered girls 2:1 at 5–10 years. The numbers of boys and girls were similar at age 11, while at 12 years there were 3.8 girls to every boy. The proportion of study children living in neighbourhoods ranked most deprived (43.4%) was twice the national average. 61.5% of children self-poisoned, 50.6% of them by ingesting analgesics. Of children who self-injured, 45.0% self-cut/stabbed, while 28.9% used hanging/asphyxiation. 32% of the children had a repeat hospital presentation for self-harm, 13.5% re-presented within a year.

**Conclusions:**

Gender patterns of self-harm until age 11 years are different to those of adolescents, with a male preponderance, especially in 5–10 years, and hanging/suffocation being more common. The frequent use of self-poisoning in this age group highlights the need for public health messages to encourage safer household storage of medicines. Self-harm in children is strongly associated with socioeconomic deprivation; understanding the mechanisms involved could be important in effective prevention.

## Background

It is recognised that self-harm occurs in children but there has been relatively little attention to this problem in the very young. Several decades ago Pfeffer and colleagues investigated self-harm and suicidal ideation in ‘latency age’ (6–12-year old) children admitted to psychiatric wards in the USA, highlighting the fact that the prevalence of suicidal behaviours (thoughts, threats or attempts) was higher in boys; 37/50 (74%) of boys compared to 5/8 (62%) of girls showed some form of suicidal behaviour [1]. This pattern was subsequently replicated in 6–12-year olds receiving out-patient treatment for a psychiatric condition [2]. This gender pattern is in marked contrast to that generally found in young adolescents who present to general hospital following self-harm, in which girls greatly outnumber boys [3]. There have been few studies of children presenting to general hospitals following self-harm. Reporting on data from a national register on hospital admissions in Australia, Mitchell and colleagues recently showed that three-quarters of individuals aged 6–10 years who presented with self-harm were boys [4]. Studart-Botto et al. [5] described self-injurious behaviour in a large population of Brazilian children under 10 years who were registered in hospital systems between 1996 and 2018. In their 5–9-year-old children, boys outnumbered girls.

In terms of methods used for self-harm, Pfeffer et al. [1] found that in children receiving care on a psychiatric ward 38% of those who self-harmed had jumped, 25% self-poisoned, 23% had burnt themselves and 13% had stabbed themselves. In the Australian study of Mitchell and colleagues [4] of hospital admissions with self-harm, self-poisoning was the most common method, as was the case in the study involving hospital registered self-harm of children under 10 years in Brazil [5].

Suicide is extremely rare in young children. However, Sheftall and colleagues in the USA, analysing data across several States, were able to identify 87 deaths of children under 12 years of age. Compared with 12–14-year olds who died by suicide, individuals in the youngest group were more often male, black, died from hanging/strangulation/suffocation, and had problems with family and friends. They were less likely to leave a suicide note, be depressed or experience problems with a boy/girlfriend [6].

There has been very little attention to self-harm in young children in the UK. Hawton et al. [7] investigated self-harm and its outcomes in children and adolescents under 15 years of age in the Oxford area between 1978 and 2003, but few were under 12 years. Of the overall group most had taken overdoses, especially of paracetamol, few appeared to have psychiatric disorders, their suicidal intent was relatively low and the most frequent problems which precipitated self-harm were difficulties in relationships with family members and school and study problems. Long-term risk of suicide was very low.

In this report, we describe the characteristics and outcomes of children aged 12 years and younger who presented to hospital for self-harm in the Multicentre Study of Self-harm in England over a 17 year period. The overall aim was to describe the characteristics of very young children who self-harm to assist clinicians who work with such populations.

## Methods

### Study design and population

The study population was identified through the Multicentre Study of Self-harm in England [8]. The Multicentre Study is a collaboration between three research centres (Oxford, Manchester, and Derby), in which data are systematically collected on all presentations for self-harm to the emergency departments in five large general hospitals, one in Oxford, three in Manchester and one in Derby. Data collection began in 2000 and is ongoing. This includes patients who do not receive a psychosocial assessment as well as those that do (in Manchester data collection on non-assessed patients began in September 2002). Information on demographic and clinical characteristics is collected on all patients who receive psychosocial assessments by specialist psychiatric practitioners in the general hospital (also by emergency department staff in Manchester). For patients who present to hospital for self-harm but do not receive a psychosocial assessment information on demographic characteristics, the method of self-harm and admission to hospital is extracted by trained researchers from emergency department electronic records.

The present analysis is based on children aged 12 years and under who had presented to the hospitals participating in the Multicentre Study of Self-harm after non-fatal self-harm for the first time between 1st January 2000 and 31st December 2016. The characteristics and outcomes of this cohort are described.

Self-harm is defined as any intentional non-fatal act of self-poisoning or self-injury, irrespective of the type of motivation, including degree of suicidal intent [9, 10]. Self-poisoning includes the intentional ingestion of any drug in an amount that is more than that prescribed and consumption of non-ingestible substances. Overdoses of ‘recreational drugs’ and severe alcohol intoxication are included only where clinical staff considers such cases to be an act of intentional self-harm. Self-injury is defined as any injury that has been intentionally self-inflicted. Inhalation of gas was classified as a method of self-injury, rather than self-poisoning, due to variations in the classification of this method in the three centres.

We distinguished between presentations to hospital which involved self-poisoning alone, regardless of the number of substances involved, and presentations which were due to self-injury alone. Presentations involving self-injury and self-poisoning together were treated as a separate group. Subsequently, we distinguished between presentations involving specific method of self-injury (based on the primary method as above): self-cutting or stabbing, jumping from heights, hanging or asphyxiation, traffic-related self-injury, and other methods (e.g. drowning). Within self-poisoning we distinguished between antidepressants, analgesics (paracetamol and salicylate in their pure or compound format), benzodiazepines (anxiolytics), major tranquilizers (antipsychotic medications), other combinations [e.g. opiates, non-steroidal anti-inflammatory drugs (NSAIDS), other sedatives], unknown drugs and non-ingestible substances.

Level of socioeconomic deprivation was based on the English Index of Multiple Deprivation (IMD). IMD is a measure of deprivation of small geographical areas in England which is derived from combining the score from several domains (income and employment, health and disability, education, skills and training, barriers to housing and services, living environment and crime). These small geographical areas are ranked according to deprivation score. The populations in the catchment areas of the centres involved in the Multicentre Study have distinctly different profiles in terms of the extent of deprivation. According to the 2015 IMD deprivation indices [11] Manchester was ranked 5th (most deprived), Derby 55th, and Oxford 166th. IMD score was derived from the patient’s postal address at a given presentation to hospital using GeoConvert http://geoconvert.mimas.ac.uk/help/faq.html. The IMD of patients with no valid address was recorded as missing. We classified the cohort into six categories based on cut-off scores determined by national IMD quintiles: 1st (least deprived) ≤ 8·49, 2nd 8·5–13·79, 3rd 13·8–21·35, 4th 21·36–34·17, 5th (most deprived) IMD score ≥ 34·18 https://tools.npeu.ox.ac.uk/imd/, and a group of individuals with no valid IMD score.

We obtained data on level of socioeconomic deprivation of the population in England according to IMD deciles by single year of age from the Office for National Statistics (UK) [12], from which we derived quintiles of deprivation for the entire population in England. We subsequently calculated the proportion of children aged 5–12 years in England in each of the five levels of deprivation (national quintiles). We compared the distribution of the study cohort to the distribution of children aged 5–12 years in England.

Information was recorded on whether or not patients were admitted to a medical bed and whether or not they received psychosocial assessment. Psychosocial assessment of the patient’s mental state, needs and risk by a mental health specialist is recommended by the National Institute for Health and Care Excellence (NICE) guidance for all patients presenting to hospital following self-harm [13, 14]. As part of the psychosocial assessment, clinicians record whether or not a patient experienced any of a list of problems preceding their episode of self-harm, including: relationship with boyfriend/girlfriend, relationship with family, relationship with friends or others, study problems, and also financial, housing, legal, physical health and mental health problems, bereavement and consequences of abuse (physical, emotional or sexual). Patients could have single or multiple problems recorded.

Repetition of self-harm was defined as any subsequent re-presentation for self-harm to the emergency departments of the study hospitals after the index episode (study entry). We also calculated the time-to-event as the time that elapsed from a given presentation to hospital for self-harm and the next presentation to hospital, if an individual had a further episode.

### Ethical approval

All three research sites have approvals to collect data on self-harm for their local self-harm monitoring systems and for multicentre projects. The monitoring systems in Oxford and Derby have approval from NHS research ethics committees to collect data on self-harm. Self-harm monitoring in Manchester is part of a local clinical audit system ratified by the local research ethics committee. All three monitoring systems are fully compliant with the Data Protection Act (1998) and have approval under section 251 of the National Health Service (NHS) Act (2006) to collect patient-identifiable information without explicit patient consent.

### Analysis

Comparisons between categorical variable were carried out using the *X*^2^ test or Fisher’s exact test, depending on the number of individuals included in each comparison.

We used two-sample test of proportion to compare the distribution of the two populations (the study cohort and the age-equivalent population of children in England) on level of IMD. Using two-sample tests of proportion we report the *z* and *p* values.

Using each person’s *first* recorded presentation to hospital as the index episode, we calculated the proportion of patients who re-presented to the study hospitals for non-fatal self-harm at any point during the follow-up. We also calculated the proportion of patients who re-presented to the study hospitals within 12 months of the first presentation in each calendar year. Analyses were run by gender.

Kaplan–Meier survival curves were produced to estimate the probability of a repeat self-harm presentation in males and females. Comparison was made using the log-likelihood test.

Analyses involving in-hospital clinical management of self-harm and precipitating problems were limited to data from two of the three research sites, due to incomplete data on the clinical management of self-harm in children for certain years in the third site.

Statistical analyses were carried out using Stata 14.2.

## Results

**Fig. 1:**
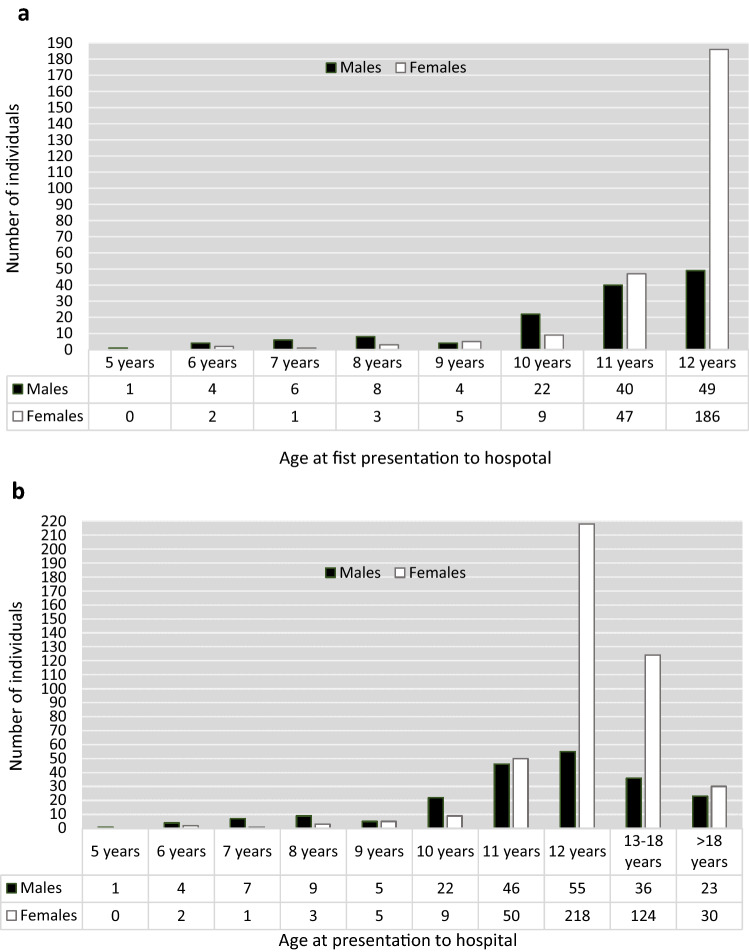
Number of children^a^ and presentations^b^ to hospital following self-harm between 2000 and 2016, by gender and age at hospital attendance. ^a^Gender and age at study entry (first presentation to the study hospitals during the study period). ^b^Gender and age at presentation to hospital—includes all episodes between 2000–2016 of individuals who were aged under 13 years at first presentation

A total of 387 children (253, 65.4% females) aged 12 years or younger at their first hospital visit after self-harm presented to the study hospitals during the 17-year study period. Age at first presentation to hospital ranged between 5 and 12 years. Over 60% (60.7%, *n* = 235) of patients were 12 years old at their first presentation to hospital, 22.5% (*n* = 87) were 11 years old, 8.0% (*n* = 31) were 10 years old, while the remaining 8.8% of patients (*n* = 34) were 5–9 years old at study entry (Fig. 1a).

There was a considerable change in the gender ratio across the age range. Between age 5 and 10 years boys outnumbered girls, with a ratio of 2.3:1. The gender ratio was approximately one at age 11 years, but at 12 years there were 3.8 girls for each boy (Fig. 1a).

These patients accounted for 650 hospital presentations for self-harm during the study period, with 437 episodes occurring at age 12 years and under and a further 213 episodes occurring when the individuals were aged 13 years and over (i.e. repeat episodes when the children were no longer under 13 years of age) (Fig. 1b).

### Socioeconomic deprivation

**Fig. 2:**
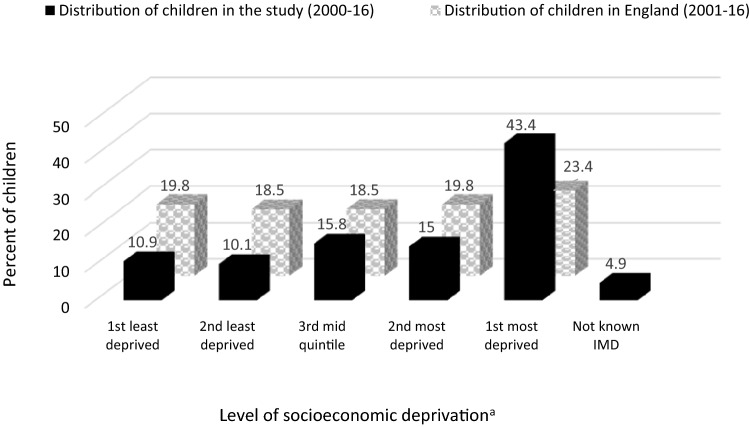
Distribution of children in the study sample according to level of socioeconomic deprivation at first hospital attendance for self-harm compared to the distribution of children in England according to national quintiles: socioeconomic deprivation is based on the English Index of Multiple Deprivation (IMD). ^a^Level of socioeconomic deprivation is based on the English Index of Multiple Deprivation (IMD) national quintiles (2001–2016)

Overall, 43.4% of children, almost twice the national proportion (23.4%), were living in areas ranked nationally as the most socioeconomically deprived at the time of their first hospital presentation (*z* = 9.3, *p* < 0.0001), while 10.9%, about half the national figure (19.8%), were living in the least deprived areas (*z* = − 4.4, *p* < 0.0001). Similarly, the proportion of children who lived in the 2nd least deprived neighbourhoods was almost half that of the age-equivalent children group nationally (*z* = − 4.3, *p* < 0.0001). There were no differences in the proportion of children living in neighbourhoods ranked 3rd quintile in terms of IMD (*z* = − 1.4, *p* = 0.16) but the proportion of children living in neighbourhoods ranked 2nd most deprived was lower than that estimated for the general population (*z* = − 2.4, *p* = 0.02) (Fig. 2).

### Methods of self-harm

More than 60% of the children (61.5%) used self-poisoning alone, 33.3% self-injury alone, and 5.2% used both methods in their first episode of self-harm. Self-injury alone was used by a greater proportion of boys (48.5%) than girls (25.3%) (*p* < 0.0001). In 5–10-year olds, the proportions of males and females who used self-injury (alone) were similar (47.7 versus 47.4%, respectively, *p* = 0.98), but in 11–12-year olds 49.4% of boys and 23.6% of girls had self-injured (*p* < 0.0001) (Table 1).

**Table 1:** Methods of self-harm in children aged 12 years and under who presented to hospital for self-harm between 2000 and 2016, characteristics at first hospital presentation

Method of self-harm	*N* (%)			*P* value
	Both genders	Males	Females	
	*N* = 387	*n* = 134	*n* = 253	
Self-poisoning alone	238 (61.5)	65 (48.5)	173 (68.4)	< 0.0001
Self-injury alone	129 (33.3)	65 (48.5)	64 (25.3)	
Self-poisoning and self-injury	20 (5.2)	4 (3.0)	16 (6.3)	
5–10-year olds				
Self-poisoning alone	33 (52.4)	23 (52.3)	10 (52.6)	0.98
Self-injury alone	30 (47.6)	21 (47.7)	9 (47.4)	
Self-poisoning and self-injury	0 (0.0)	0 (0.0)	0 (0)	
11–12-year olds				
Self-poisoning alone	204 (63.4)	41 (46.1)	163 (70.0)	< 0.0001
Self-injury alone	99 (30.7)	44 (49.4)	55 (23.6)	
Self-poisoning and self-injury	19 (5.9)	4 (4.5)	15 (6.4)	

Substances used for self-poisoning^a^	*n* = 243	*n* = 65	*n* = 178	
Analgesics	123 (50.6)	23 (35.4)	100 (56.2)	0.004
Antidepressants	19 (7.8)	10 (15.4)	9 (5.1)	0.008
Benzodiazepines	7 (2.9)	3 (4.6)	4 (2.3)	0.39
Other drugs (e.g. opiates, NSAIDS)	106 (43.6)	27 (41.5)	79 (44.4)	0.69
Non-ingestible substances	19 (7.8)	8 (12.3)	11 (6.2)	0.12

Specific methods of self-injury^b^	*n* = 143	*n* = 67	*n* = 76	
Self-cut or stab	67 (45.0)	21 (30.4)	46 (57.5)	0.001
Hanging or asphyxiation	43 (28.9)	30 (43.5)	13 (16.3)	< 0.0001
Traffic-related injuries	4 (2.7)	0 (0.0)	4 (5.0)	0.12
Jump from a high place	6 (4.0)	4 (5.8)	2 (2.5)	0.42
Other (e.g. head banging)	29 (19.5)	14 (20.3)	15 (18.8)	0.81

^a^From patients who self-poisoned and the specific medication was known

^b^From patients who self-injured

Over half of children (50.6%) who presented to hospital after self-poisoning took analgesics at their first episode, 7.8% ingested antidepressants, and 2.9% used benzodiazepines. Other drug groups involved antipsychotic and mood stabilizers, opiates and NSAIDS but these were consumed by a small proportion of young children and, therefore, recorded under ‘other medication’. Analgesics were used by more of the girls (56.2%) than the boys (35.4%) (*p* = 0.004). Of patients who self-injured, 45.0% self-cut or stabbed and 28.9% used hanging or asphyxiation. The proportion of females who self-cut was higher than that of males (*p* = 0.001) while the proportion of males who self-injured by hanging or asphyxiation was higher in males than in females (43.5 versus 16.3%, *p* < 0.0001).

### Clinical management and problems experienced by the children

In this section, we limited our analysis to two of the three research sites since information on the clinical management of self-harm in children was incomplete for certain years for one of the centres (Manchester). In the two included centres, there were 175 children aged under 13 years at their first hospital presentation for self-harm, 82.9% (*n* = 145) of whom received a psychosocial assessment at their first presentation to hospital (77.6%, *n* = 38 of males and 84.9%, *n* = 107 of females, *p* = 0.25). Furthermore, 64.6% (*n* = 113) of those children were admitted to a bed in the general hospital following self-harm (61.2%, *n* = 30 of males and 65.9%, *n* = 83 of females). Over a quarter (26.9%; *n* = 47) were not admitted while this was unknown for 8.6% (*n* = 15) of patients.

In patients who received a psychosocial assessment (*n* = 145), the problems most commonly identified as preceding their self-harm were relationships with family members (33.8%), or with friends or others (19.3%), study problems (13.1%) and problems resulting from physical, emotional or sexual abuse (7.5%), and mental health problems (6.5%) (data missing for 4% of individuals). There were no gender differences in the prevalence of specific problems.

### Repetition of self-harm

In this analysis, we limited the analytic sample to patients who presented to hospital for the first time between 2000 and 2015 (*n* = 333) to allow at least one year of follow-up for repetition. Follow-up for repetition was to the end of 2016. Thirty-two percent of individuals had a repeat presentation to hospital at any time between the index episode (the first recorded presentation to hospital in the study period while under 13 years) and 2016. Repetition of self-harm was more common in the girls (38.5%, 85/221) than the boys (18.8%, *n* = 21/112) (*χ*^2^ = 13.3, *df* = 1, *p* < 0.0001).

Overall, including repeat presentations which occurred after age 12 years, 227 (68.2%) presented to hospital once during the study period, 58 (17.4%) presented to hospital for self-harm twice, 22 (6.6%) had three presentations to hospital, with the remainder (7.8%) having four or more (maximum 17) presentations to hospital.

**Fig. 3:**
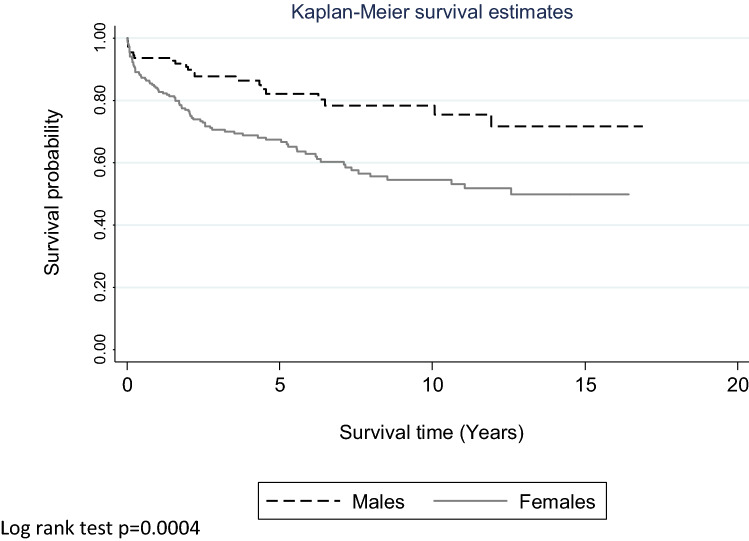
Probability of a repeat self-harm episode in males and females aged 12 years and younger after first presentation to hospital in the study period. Log rank test *p* = 0.0004

A repeat presentation to hospital for a further episode of self-harm within the first year after the index episode "(the first recorded presentation to hospital in a calendar year) occurred in 13.5% (*n* = 47) of the 348 children in the analytic sample (in this analysis a child may re-enter the analytic sample every year but they will only be included in the analytic sample if they were 12 years and under in that year). Repetition within a year of the index episode occurred in 8.5% (*n* = 7) of 11-year olds and in 17.9% (*n* = 39) of the 12-year olds. However, in children aged 5–10 years at their index episode only one individual (aged eight years) repeated within a year of their first presentation (Fig. 3).

## Discussion

We studied the characteristics of and outcomes in children aged 12 years and younger who had presented to hospital for self-harm during 2000–2016. Self-harm which results in presentation to hospital is relatively uncommon in this age group; overall, 263 children presented to the study hospitals over 17 years. The majority of these (60%) were aged 12 years at their first recorded episode.

### Gender ratio

The gender ratio changed markedly with age in these children. Between age 5 and 10 years, there were more boys than girls presenting to hospital for self-harm, but at age 11 the gender ratio was approximately one. At age 12 years there was a remarkable shift in the gender ratio, with girls outnumbering boys by nearly four to one. This finding is in keeping with a study in the USA several decades earlier in which the majority of 6–12-year-old children with suicidal behaviours or thoughts were boys [1], although the sample was of children who had been admitted to a psychiatric ward so the male predominance might have reflected differences in psychiatric morbidity between the genders or severity of self-harm acts rather than just presence of suicidal behaviour. Nevertheless, Pfeffer [2] replicated these findings in a sample of 6–12-year-old psychiatric out-patients. In a study in Australia, Mitchel et al. [4] found that 76% of the 6–10-year old in their sample of hospital admitted individuals were boys. This excess of boys was also found in a large register-based study in Brazil of admissions to hospital of under 10-year olds with self-harm [5].

The findings of our study are probably the most detailed and accurate that have been recorded for the UK on self-harm in children. Previously, studies have largely focussed on adolescents. The Office for National Statistics recently released national data on self-harm admissions to hospitals in under 13-year olds in England [15]. However, the fact that these data included a considerable number of children aged four years and under suggests that some children are likely to have been misclassified as self-harming rather than accidental poisoning or injuries since an act of harm by a child under 5 years cannot be reliably interpreted as self-inflicted harm and, therefore, the findings may be somewhat misleading. The data did, nevertheless, as in our study, show a male excess in children aged 6–10 years recorded as self-harming. Thus predominance of boys among children self-harming seems to be the general pattern.

The abrupt increase in girls self-harming at age 12 years was striking. It may well reflect the onset of puberty and its recognised association with increased risk of self-harm in girls [16, 17] although a study involving more than 1000 under 13-year-old community-based non-treatment seeking children in Australia has shown that past year self-harm at age 11–12 years was more likely in children who were in mid-late puberty or late puberty relative to early puberty [18]. Other relevant factors might include starting secondary education, although one would perhaps expect similar effects in boys.

### Socioeconomic deprivation

Our data showed an association between socioeconomic deprivation and self-harm, with 43.4% of the under 13-year olds living in areas ranked as the most socioeconomically deprived. The distribution of children in the study was different from that of the age-equivalent children in England, with a greater proportion of children residing in neighbourhoods ranked nationally as most deprived. Interestingly, this was also the pattern found in Mitchell and colleagues’ study of hospitalised children and adolescents in Australia [4]. Numerous studies have demonstrated an association between socioeconomic deprivation and self-harm as well as suicide in adults [19] and also in adolescents [20–22], but data on children under 13 years are limited. Interestingly, Mok et al. [22] studied self-harm which occurred in individuals aged 15–33 years but the points of exposure to socioeconomic adversity were the parents’ socioeconomic position at the offspring’s birth year and then at age 5, 10 and 15 years. Parental income at any point measured during childhood or adolescence was inversely associated with risk of self-harm during late adolescence and young adulthood (15–33 years). Furthermore, the length of time a child lived in poorer conditions was related positively to self-harm risk. These and our findings indicate that socioeconomic deprivation plays an important role in the early engagement of children in self-harm. Further work on the pathway linking deprivation and self-harm in children is warranted as this will have direct implications for primary and secondary prevention.

### Self-harm methods

The majority of children presented to hospital after self-poisoning alone, primarily with analgesics. Analgesics were taken in overdose more often by females, while males more commonly overdose of antidepressants. Children who self-injured did so mainly by cutting or stabbing themselves. Of particular concern is the fact that the second commonest method of self-injury was attempted hanging or suffocation, with 28.9% using this method. While self-cutting was more common in the girls, a substantial proportion of the boys attempted hanging or suffocation.

### Clinical management and problems

Using data from two of the three research sites, we found that at first presentation to hospital for self-harm 83% of the children received a psychosocial assessment and 65% were admitted to a medical bed in the hospital. There were no gender differences in the proportion of children assessed and admitted. This shows that not all children receive the care specified by national guidance [13] that children who present to hospital for self-harm should receive a psychosocial assessment and should be admitted to a medical bed, although in some of the cases where individuals left hospital before having a psychosocial assessment this may have been due to parents discharging their children before an assessment could take place, or occasionally an assessment being arranged outside hospital.

Our finding that problems with family members and peers are commonly cited problems in children who self-harm is in line with earlier reports from Australia [18] and the USA [6]. This adds to an extensive body of literature about the link between thwarted social ties and self-harm in children and adolescents [23, 24].

### Outcomes

Almost a third of children had a further presentation to hospital during the study period, including presentations when they were aged over 12 years. Fourteen percent had a repeat episode within a year, which is a lower proportion than in adolescents [25]. However, risk of repetition within a year of a first presentation was strongly associated with age, with twice as many 12-year olds repeating self-harm than 11-year olds, and only one of those aged 5–10 years repeating. Girls were considerably more likely than boys to repeat self-harm.

### Strengths and limitations

The study was based on data collection in three centres with varying characteristics, including socioeconomic deprivation, across a substantial period of time. Therefore, the findings are likely to be reasonably representative of children who present to hospitals with self-harm in England. The study is, however, solely focussed on individuals presenting to hospital following self-harm. It is well known that much larger number of adolescents self-harm than present to hospital [26]; the same is likely to be true for children. There may be an increased likelihood that self-harm episodes in children, especially the very young, are not recognised as such, perhaps being more likely than in older individuals to be regarded as accidental. Furthermore, our finding that the distribution of children in the study in terms of socioeconomic deprivation was different from that of the age-equivalent children in England, with a greater proportion of children residing in neighbourhoods ranked nationally as most deprived may have reflected, at least in part, the fact that the catchment areas of the study hospital are predominantly urban.

In addition, we do not have information on psychiatric diagnoses. Information about diagnoses and their relationship with outcomes such as self-harm repetition would be of great value for clinical practice and therefore would be an important area for further research in very young individuals who self-harm.

Our data on hospital care for children was limited to information provided by two of the three research sites. The generalizability of our findings on the proportion of children receiving psychosocial assessment or admission to hospital may be limited.

### Clinical implications

This study shows that while self-harm is relatively uncommon in children, at least up to age 11 years, it is nevertheless important in this age group. The pattern appears to be somewhat different to that in young adolescents, with a preponderance of males up to age 10 years and similar numbers of males and females at age 11 years before a marked switch to considerably more females than males at age 12 years (a roughly four to one ratio), a phenomenon as noted above that may be associated with onset of puberty [16].

It is important that clinicians involved in assessment and interventions with children who may self-harm are aware of this pattern, and especially the frequency of self-injury—particularly hanging and suffocation, most notably in boys. Repetition of self-harm in this study was frequent in the longer term and some of the methods of self-harm used were clearly very dangerous, raising the possibility of suicide, with such deaths not being unknown in this age range [6].

The relatively frequent use of self-poisoning in this age group highlights the need for public health messages to encourage safer storage of medicines in households, as the drugs used were most likely to have been from domestic sources.

Our data suggest that not all children receive the NICE recommended care after presentation to hospital for self-harm. A concerted effort is required to improve these figures; some of this effort may need to include an investigation into the reasons for lack of assessment and admission.

## Conclusions

Self-harm in children, while far less common than in adolescents, is nonetheless an important clinical problem. Gender patterns, at least until age 11 years, are very different to those of adolescents, with a male gender preponderance, especially in the very young. Also, there is considerable use of self-injury, including attempted hanging and asphyxiation. Self-harm in children has a strong association with socioeconomic deprivation.

## Data Availability

We have approval under section 251 of the NHS Act (2006) to collect patient-identifiable information without patient consent. This does not allow us to share the data.

## Abbreviations

IMD: Index of Multiple Deprivation
ONS: Office for National Statistics
NICE: National Institute for Health and Care Excellence
